# Supplementary material for: Tuning LASSO Models for Propensity Score Weighting and Using Synthetic Negative Control Exposures for Residual Bias Detection
Source: Stat Med. 2026 Apr 7;45(8-9):e70503. doi: 10.1002/sim.70503 (PMC13055431; doi:10.1002/sim.70503)
Supplement: Supplementary file 1 — Data S1: Supporting Information. [file SIM-45-0-s001.pdf]

S1 Supplemental Appendix

S1.1 General Overview of the Highly Adaptive LASSO

The Highly Adaptive Lasso (HAL) is a machine learning prediction algorithm that can be used to nonparametrically estimate regression functions. To understand HAL, it is necessary to understand the conditions (or assumption) required by HAL. First, HAL requires the total variation of a function (or variation norm) to be bounded, where the total variation can be thought of as a measure of the complexity of a function. For example, in the simple case of a monotone function, $f(\cdot)$, on the interval $[0, 1]$, the total variation is simply $|f(1) - f(0)|$. For more general functions, the total variation is equal to the cumulative sum of all the absolute incremental changes in the function's values over its domain. Intuitively, if we plotted a non-linear/non-monotone continuous univariate function and traced the function with a piece of string, the total variation of the function can be thought of as the length of the string. Being able to bound the total variation of a function ensures that the function cannot wiggle around too much. The concept of bounded total variation can be extended to higher dimensions (and non-continuous functions), but the general idea remains the same.

By requiring the total variation of the function to be bounded, HAL places a global constraint on the behavior of the function rather than local constraints. This distinction is important to understanding HAL. The former controls how much the function can fluctuate globally, while the latter only controls how much the function can fluctuate locally at each point on its domain. One can globally constrain a function by imposing very strong local smoothing constraints; for example, by requiring the function to be many times differentiable with bounded derivatives. However, as the dimension grows, the smoothness constraints needed to globally constrain the function become very strong. This leads to the curse of dimensionality where conventional methods fail to estimate high dimensional functions at a fast enough rate. The main challenge for local smoothing methods is that they try to impose a global constraint on the function by imposing increasingly restrictive local constraints. HAL circumvents this issue by imposing a global constraint directly without imposing any local smoothness constraints.

In addition to requiring the total variation to be bounded, HAL requires some regularity conditions on the function. Mainly, that the function is cadlag, meaning that it is mostly continuous everywhere but can jump finitely many times. Cadlag functions are very general and do not require local smoothness (allow discontinuities). Understanding this function class is also key to understanding HAL since the implementation of HAL is based on recognizing two features of cadlag functions of bounded total variation. First, they can be approximated arbitrarily well by linear combinations of indicator jump functions (e.g., $\mathbb{1}(X \geq x)$). Second, the total variation (or variation norm) of a linear combination of indicator jump functions is equal to the absolute sum of the regression coefficients in front of the indicator jump functions.

In the context of a propensity score function, $e(X)$, that is cadlag with a bounded variation norm, Benkeser & van der Laan (2016) show that the baseline covariates, X, can be expanded into a series of $n(2^d - 1)$ binary indicator variables (i.e., indicator basis functions), W, such that as $n \to \infty$ the logit of the propensity score function, $logit(e(X))$, can be approximated arbitrarily well by a linear combination of the binary indicators written as:

$$logit(g(W)) = \gamma_0 + W^\top \gamma, \tag{4}$$

where $g(W) = P(A|W)$, γ is a $n(2^d - 1)$ dimensional vector of parameters, and γ_0 is a scalar. For a theoretical explanation on the construction of the indicator basis functions, W, see Benkeser & van der Laan (2016) and Ertefaie et al (2022).

In real world settings with finite sample size, it is not possible to adjust for the entire set of binary features in W. Consequently, some dimension reduction is needed to approximate $g(W)$. Benkeser and van der Laan (2016) show that LASSO regression serves this purpose through regularization and provides theoretical guarantees on fast convergence rates. As a result, HAL simply defines the optimization problem as a LASSO regression over the transformed binary indicators, W. In the context of estimating the PS, HAL becomes an L1-regularized logistic regression, where the

parameter vector, γ, in Equation 4 is estimated based on the following penalized likelihood:

$$\mathcal{L}(\gamma) = \sum_{i=1}^{n} -A_i log(p(A_i|W_i);\gamma) - (1-A_i)log(1-p(A_i|W_i;\gamma)) + \lambda \sum_{j=1}^{m} \mid \gamma_j \mid \quad (5)$$

where n is the sample size and m is the number of parameters in γ, which can be up to $n(2^d - 1)$. As $n \to \infty$, HAL will converge to the logistic model defined in Equation 4, but in finite samples HAL is just an approximation to $g(W)$.

S1.2 Proof of Proposition 1

Proposition 1: Assume we have the data structure, $\{Y, X, A, Z\}$, where Z is a negative control exposure that satisfies Definition 1. For any $X_s \subseteq X$, if $(Y^{a=1}, Y^{a=0}) \perp\!\!\!\perp A|X_s$ then $Y \perp\!\!\!\perp Z|X_s, A$.

Proof. Let X_s be any subset of X such that $Y^a \perp\!\!\!\perp A|X_s$. The proportional odds assumption in Defininition 1 ensures that $P(Z|X)$ can be written as a 1-to-1 function of $P(A|X)$. This implies that all predictors of Z and A are equivalent. Therefore, for any variable not in X_s that is a common cause of Z and Y, that variable's effect on Y must be mediated through the exposure, A. Otherwise, X_s would not be sufficient to satisfy $Y^a \perp\!\!\!\perp A|X_s$. This implies that X_s is sufficient to satisfy $Y^z \perp\!\!\!\perp Z|X_s, A$. From the definition of a negative control exposure, we further know that $Y^{(a,z)} = Y^a$ (no causal effect of Z on Y). This further implies that $Y^{z=0} = Y^{z=1} = Y$. Therefore, $Y \perp\!\!\!\perp Z|X_s, A$. □

S1.3 Proof of Proposition 2

Proposition 2: Assume we have the data structure, $\{Y, X, A, Z\}$, where Z is a negative control exposure that satisfies Definition 1. For any $X_s \subseteq X$, if $Y^{a=0} \perp\!\!\!\perp A|X_s$ then $Y \perp\!\!\!\perp Z|X_s, A = 0$.

Proof. Let X_s be any subset of X such that $Y^{a=0} \perp\!\!\!\perp A|X_s$. From the definition of a negative control exposure, $Y^a \perp\!\!\!\perp Z|X_s, A$ and $A \perp\!\!\!\perp Z|X_s$. This implies that $Y^{a=0} \perp\!\!\!\perp Z|X_s, A = 0$. Therefore, $Y \perp\!\!\!\perp Z|X, A = 0$ by consistency. □

S1.4 Further Discussion of the Proportional Odds Assumption

The goal of the proportional odds assumption is to have covariate differences across negative control exposure groups within the unexposed that approximate covariate differences across the exposure groups in the full population. If covariate differences across exposure groups match those across negative control exposure groups within the unexposed, then under the assumption that the association between X and A is linear, it can be shown that the magnitude of confounding bias caused by X on the relationship between A and $Y^{a=0}$, will be equal to the bias caused by X on the relationship between Z and the observed outcome, Y, among the unexposed on the absolute scale (see formal description of this statement and proof below). If the relationship between X and A is not linear, but is linear in some expanded basis (e.g., the Highly Adaptive Lasso), then the same arguments hold and are generally applicable by replacing X with the expanded set of basis functions. In practice, however, the proportional odds assumption only guarantees that covariate differences across negative control exposure groups within the unexposed will approximate differences across exposure groups in the full study population; they will not be exact.

Assume we have the data structure defined above, where $Y^{a=0} \perp\!\!\!\perp A|X$ and assume the relationship between X and A is linear (or linear on the log-odds scale). Let $D_1 = E[X|A = 1] - E[X|A = 0]$ represent the mean difference in the covariates, X, across exposure groups and let $D_2 = E[X|Z = 1, A = 0] - E[X|Z = 0, A = 0]$ represent the mean difference in X across synthetic exposure groups after restricting on $A = 0$. Further, let $B_1 = E[Y^{a=0}|A = 1] - E[Y^{a=0}|A = 0]$ represent the bias caused by X on the effect of A on $Y^{a=0}$ on the risk difference scale (bias in the unadjusted risk difference), and let $B_2 = E[Y|Z = 1, A = 0] - E[Y|Z = 0, A = 0]$ represent the bias caused by X on the effect of Z on Y within the unexposed population on the risk difference scale (bias in the synthetic unadjusted risk difference). If $D_1 = D_2$ it follows that $B_1 = B_2$.

Proof. Let $Y^{a=0} \perp\!\!\!\perp A|X$. Using the generalized bias formulas for uncontrolled confounding developed by Vanderweele & Arah (2011), the magnitude of bias caused by X on the effect of A on $Y^{a=0}$

on the risk difference scale can be expressed as

$$\sum_x \{P[Y^{a=0}|A = a, X = x] - P[Y^{a=0}|A = a, X = x']\}\{P(X|A = 1) - P(X|A = 0)\}P(X = x)$$

If we make the simplifying assumption that the relationship between X and A is linear (or linear on the log-odds scale), the this implies that $P(X|A = 1) - P(X|A = 0)$ does not vary between strata of X. Under this condition, Vanderweele & Arah (2011) show that the above expression then simpifies to

$$\{P[Y^{a=0}|A = a, X = x] - P[Y^{a=0}|A = a, X = x']\}\{P(X|A = 1) - P(X|A = 0)\}$$

Linearity between X and A also implies that the above expression further reduces to

$$\{P[Y^{a=0}|A = a, X = x] - P[Y^{a=0}|A = a, X = x']\}\{E(X|A = 1) - E(X|A = 0)\}$$

Similarly, the bias caused by X on Z and Y among the unexposed (i.e., those with $A = 0$) can be expressed as

$$\{P[Y|X = x, A = 0] - P[Y|X = x', A = 0]\}\{E(X|Z = 1, A = 0) - E(X|Z = 0, A = 0)\}$$

The last two expressions are equal since $P[Y|X = x, A = 0] = P[Y^{a=0}|X = x, A = 0] = P[Y^{a=0}|X = x]$ and we assume that $E(X|Z = 1, A = 0) - E(X|Z = 0, A = 0) = E(X|A = 1) - E(X|A = 0)$. $\square$

S1.5 Supplemental Figures

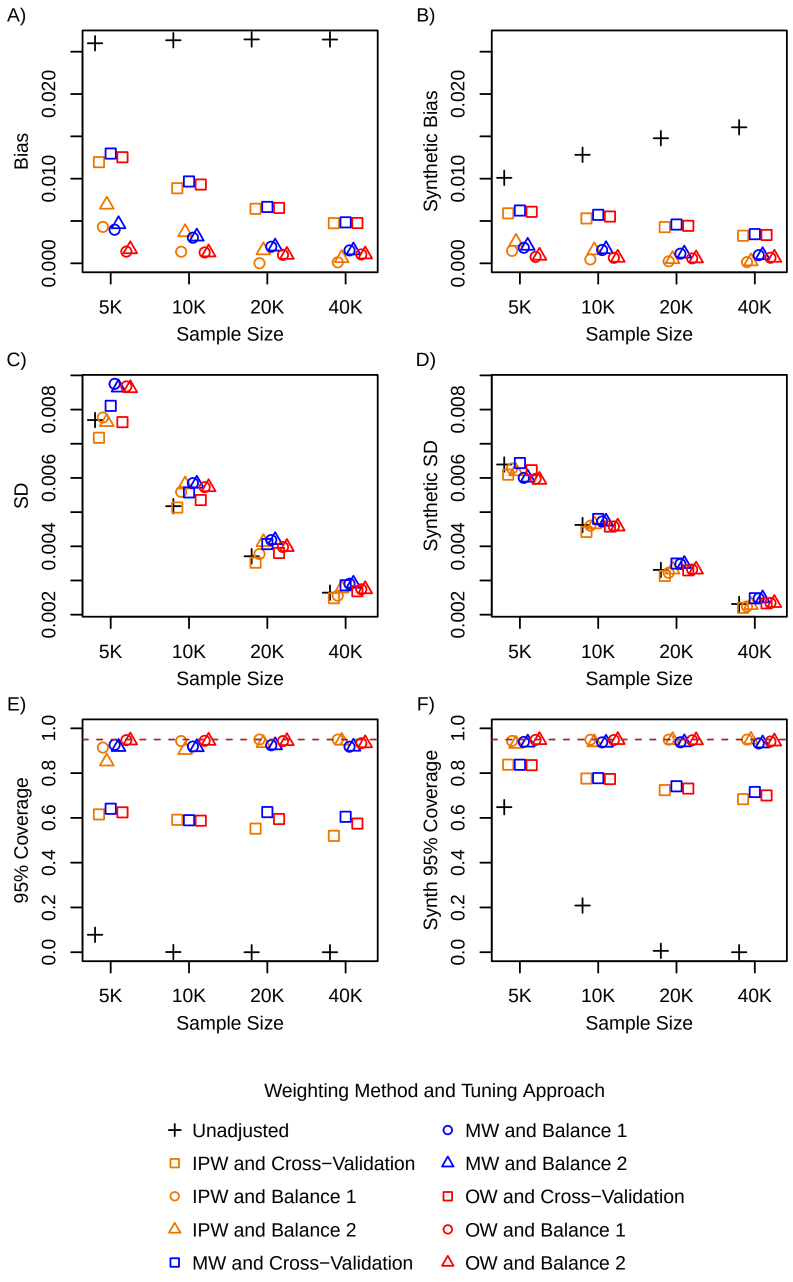

Supplemental Figure 1: Bias, standard deviation (SD), and 95% coverage probabilities in effect estimates (Plots A, C, and E) and synthetic negative control effect estimates (Plots B, D, and F) for Simulation Setup 1.

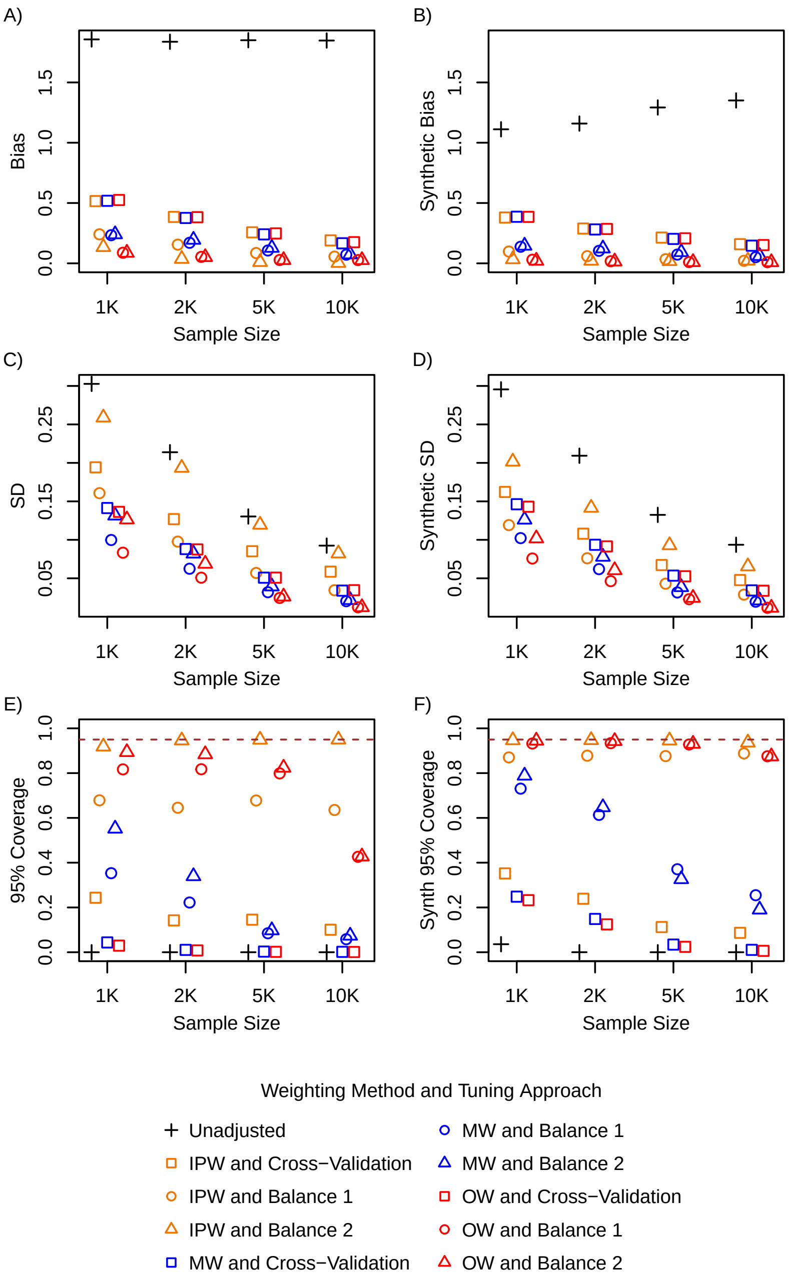

Supplemental Figure 2: Bias, standard deviation (SD), and 95% coverage probabilities in effect estimates (Plots A, C, and E) and synthetic negative control effect estimates (Plots B, D, and F) for Simulation Setup 2.
